# Supplementary material for: BCAT1 controls metabolic reprogramming in activated human macrophages and is associated with inflammatory diseases
Source: Nat Commun. 2017 Jul 12;8:16040. doi: 10.1038/ncomms16040 (PMC5510229; doi:10.1038/ncomms16040)
Supplement: Supplementary Information [file ncomms16040-s1.pdf]

Title of file for HTML: Supplementary Information
Description: Supplementary Figures, Supplementary Methods and Supplementary References

Title of file for HTML: Peer Review File
Description:

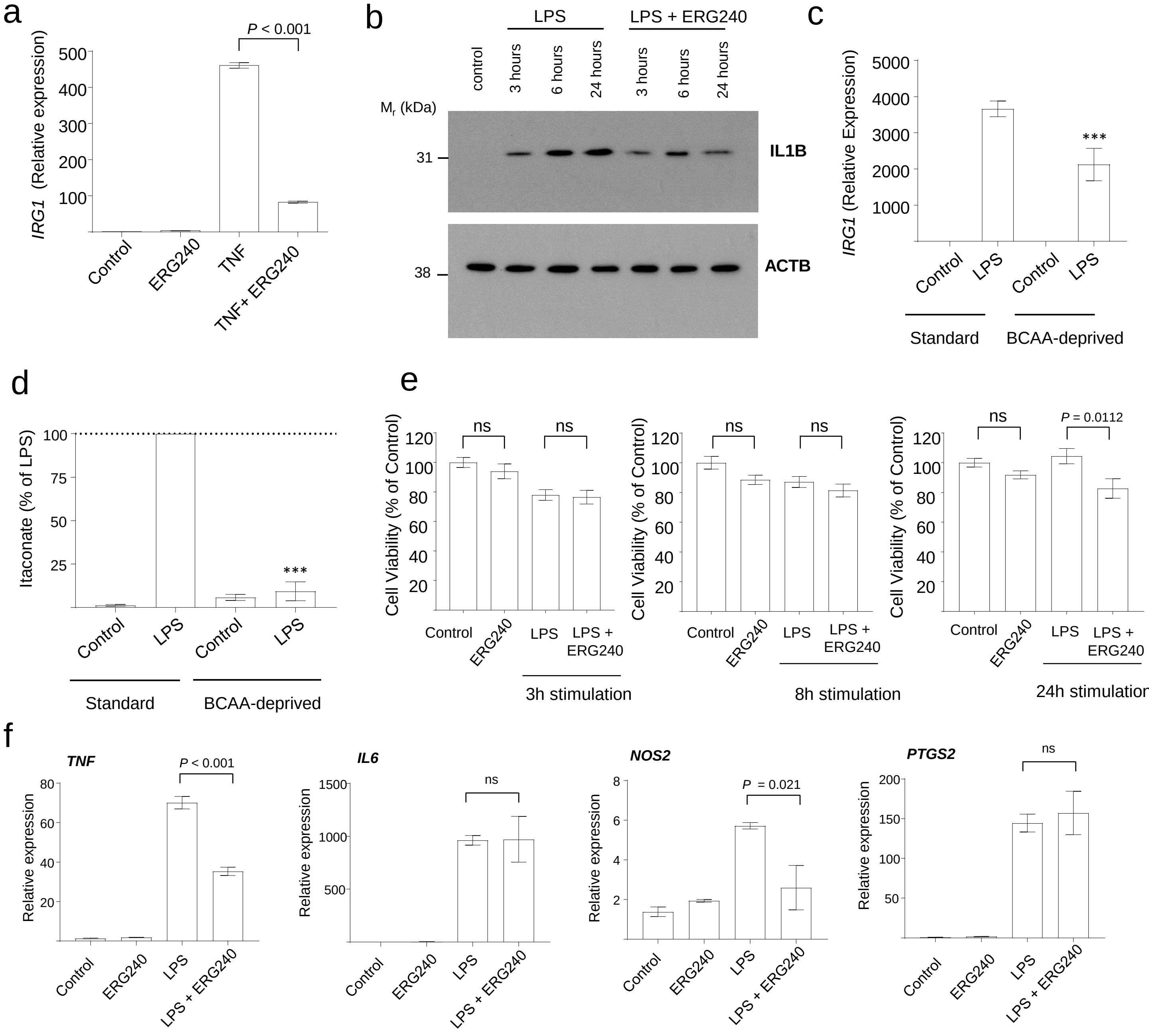

Supplementary Figure 1. (**a**) Relative expression of *IRG1* (normalized to *ACTB*) by qRT-PCR in M(basal), ERG240-treated (20mM, 3hours), TNF treated (2 ng/ml, 8h) and TNF+ERG240 treated (TNF, 2 ng/ml; ERG240, 20 mM for 8 hours) hMDMs. **(b)** Western Blot analysis of IL1B and ACTB in control (untreated), LPS treated (100 ng/ml; 3h, 6h, 24h) and LPS+ERG240 treated (LPS, 100 ng/ml; ERG240, 20 mM; 3h, 6h, 24h) hMDMs. (**c-d**) hMDMs were incubated with standard or BCAA-deprived conditioned media for 8 hours in either basal or LPS-treated (100 ng/ml) conditions. *IRG1* expression and itaconate levels were measured by qRT-PCR (normalized to *B2M*) (**c**) and GC/MS (**d**), respectively. (**e**) Cell viability in hMDMs treated with either LPS (100ng/ml) or ERG240 (20mM) or both at 3h, 8h and 24h stimulation periods. **(f)** Relative expression of *TNF*, *IL6*, *NOS2* and *PTGS2* (normalized to *HPRT*) measured by qRT-PCR in M(basal), ERG240-treated (20mM, 3hours), LPS treated (100ng/ml, 3h), and LPS+ERG240 treated (LPS, 100 ng/ml; ERG240, 20 mM for 3 hours) hMDMs. At least, n=3 donor hMDMs were used. Error bars are s.e.m. Significance was tested using one-way ANOVA. ***, $P < 0.001$ when compared with LPS standard by one-way ANOVA; ns, non-significant.

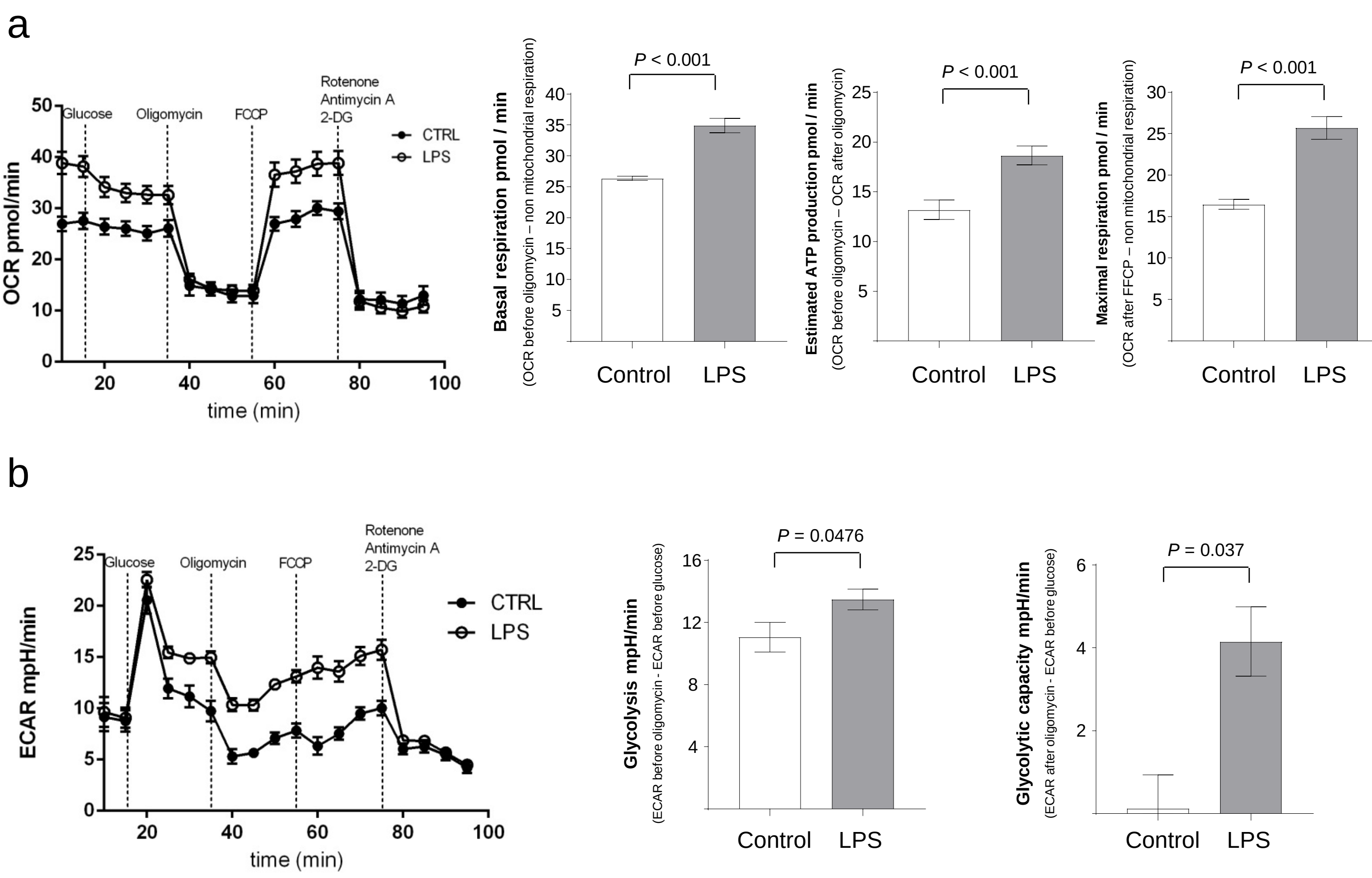

Supplementary Figure 2. Early LPS effect on oxygen consumption rate (OCR) and extracellular acidification rate (ECAR) in human MDMs. Real-time extracellular OCR (**a**, left panel) and ECAR (**b**, left panel) measurements in hMDMs that were previously treated with LPS (100 ng/ml, 3h). Control (CTRL, untreated) cells were used for comparison and OCR values were recorded following addition of glucose, oligomycin A, FCCP and a combination of 2-DG, antimycin A and rotenone. Right panels show basal respiration, estimated ATP production, maximal respiration for OCR; glycolysis and glycolytic capacity for ECAR in CTRL and LPS-treated hMDMs. n=3 healthy donor hMDMs were used in 12 technical replicates. Error bars are s.e.m. Significance was tested using two-tailed Student’s t-test. The results are representative of three independent experiments.

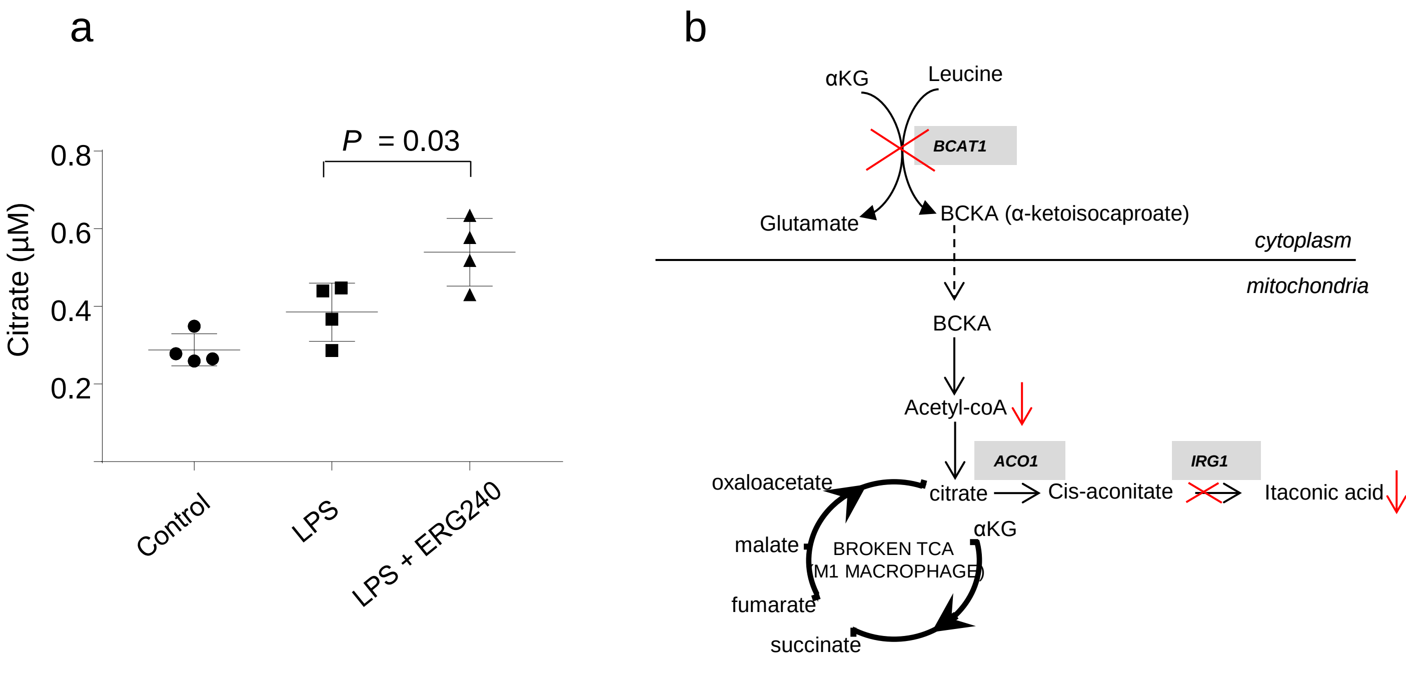

Supplementary Figure 3. Citrate levels and schematic illustration of BCAA pathway in human macrophages. (**a**) Citrate concentrations were measured by GC-MS in basal-, ERG240-, LPS-, and LPS+ERG240 treated hMDMs (n=4 donors). LPS treatment was for 3 hours at 100ng/ml. (**b**) Schematic representation of the interaction between BCAA catabolism and the broken Krebs cycle. The arrows do not take into account the reversibility of enzymatic reactions. BCAT1 and the TCA enzymes of the citrate break are shown in grey. The red cross and arrows indicate the possible sites impacted by ERG240. Error bars are s.d. Significance was tested using one-way ANOVA.

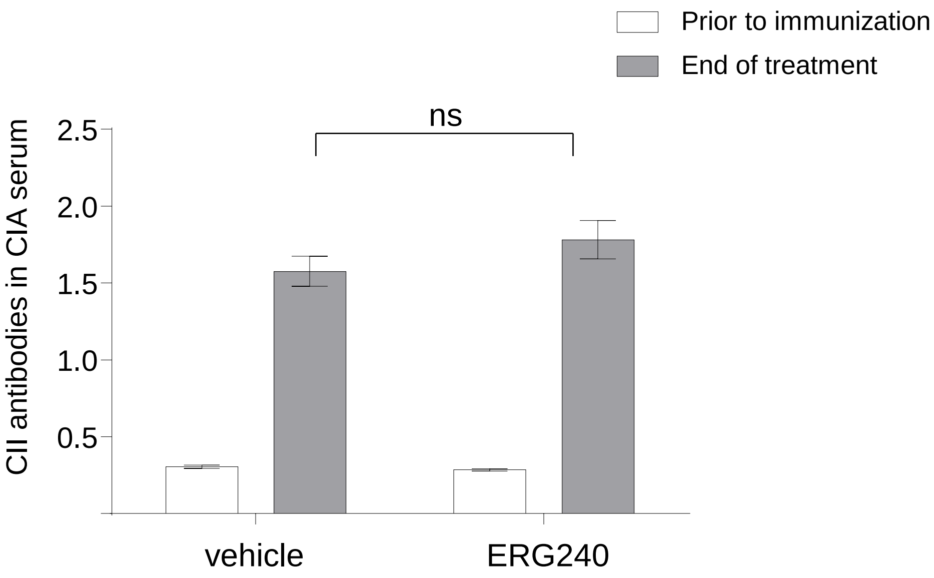

Supplementary Figure 4. Levels of circulating anti-type II collagen antibody prior to immunization and at the end of the therapeutic experiment for indicated groups (vehicle and ERG240). Error bars are s.e.m. Significance was tested by one-way ANOVA. ns, non-significant.

a

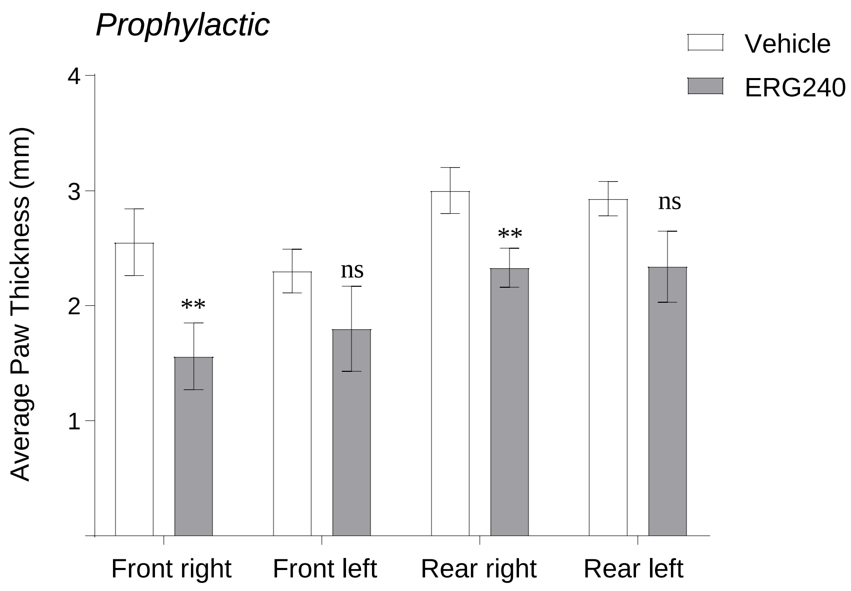

b

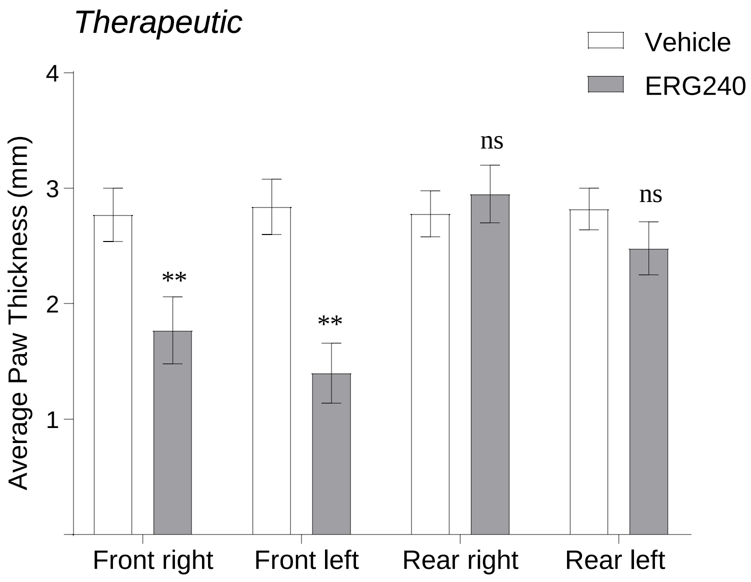

Supplementary Figure 5. Terminal thickness of fore and hind paws of animals treated (**a**) prophylactically or (**b**) therapeutically. Error bars are s.e.m. *, *P* < 0.05; **, *P* <0.01 by two-tailed Student's t-test; ns, non significant.

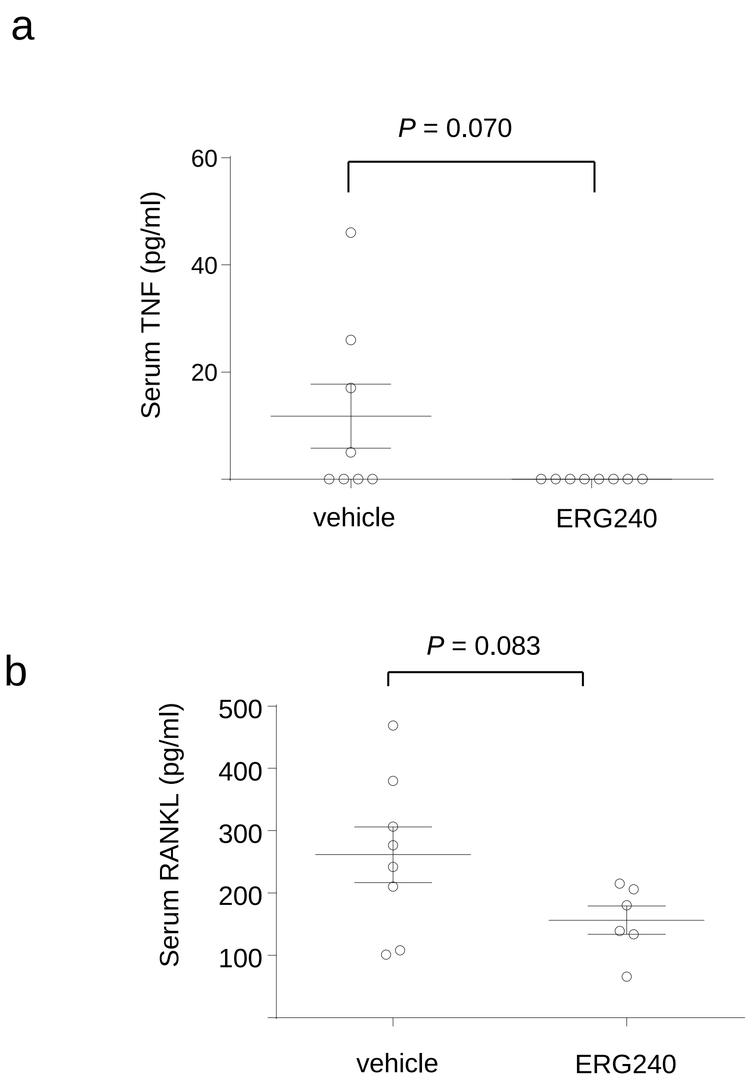

Supplementary Figure 6. ERG240 therapeutic treatment in CIA and circulating levels of (**a**) TNF, (**b**) RANKL measured by Sandwich ELISA. Error bars are s.e.m. Significance was tested using two-tailed Student's t-test.

Primer sequences

Human :

IRG1-F TTCCATGAATGCCAGATCAA
IRG1-R GGTTTTCTCCAGTGCCCATA

TNF-F TCCTTCAGACACCCTCAACC
TNF-R AGGCCCCAGTTTGAATTCTT

HIF1A-F TCCAAGAAGCCCTAACGTGT
HIF1A-R TGATCGTCTGGCTGCTGTAA

BCAT1-F TGAGGCTTGGCTTTTGTGAA
BCAT1-R GGCTCTGGTGTAACAAAGCC

B2M-F TTAGAGGTGGGGAGCAGAGA
B2M-R TCCCCCAAATTCTAAGCAGA

ACTB-F AGAGCTACGAGCTGCCTGAC
ACTB-R AGCACTGTGTTGGCGTACAG

NOS2-F CTCTATGTTTGCGGGGATGT
NOS2-R TTCTTCGCCTCGTAAGGAAA

PTGS2-F TACCCTCCTCAAGTCCCTGA
PTGS2-R ACTGCTCATCACCCCATTCA

IL1B-F ACTGAAAGCTCTCCACCTCC
IL1B-R CTCTCCAGCTGTAGAGTGGG

HPRT-F TGAGGATTTGGAAAGGGTGT
HPRT-R AATCCAGCAGGTCAGCAAAG

IL6 -F AGTCCTGATCCAGTTCCTGC
IL6 -R AAGCTGCGCAGAATGAGATG

Mouse:

Irg1-F GGACCCTCTGTCCAAGATGA
Irg1-R CTTTGCTGAGCTCATTGCTG

Rat:

Col1a1-F AACAAGGGAGGAGAGAGTGC
Col1a1-R TGGTGCTCTGAAACCCTGAT

siRNA sequences for human *BCAT1*

1. GUACAAAGGCGAGACAAUA
2. GAAGGCAUUUCGAGGAGUA
3. GGAGUGGUCCUCAGAGUUU
4. GAAGAGAGCGACUGGACAA

Supplementary Figure 7. Primer and siRNA sequences used.

Fig 1e

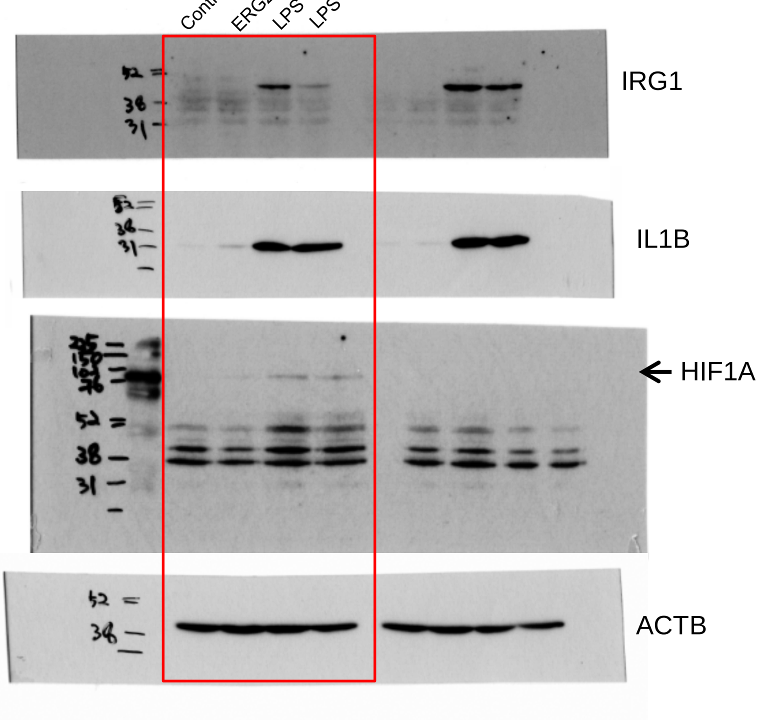

Fig 3a

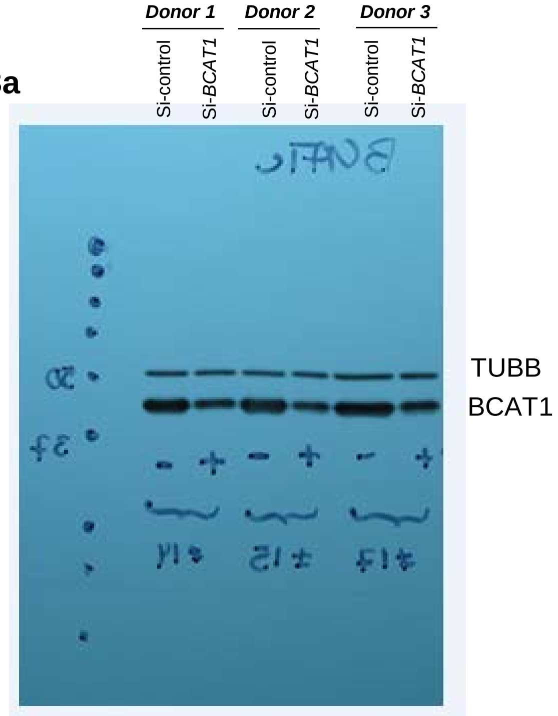

Fig 3a

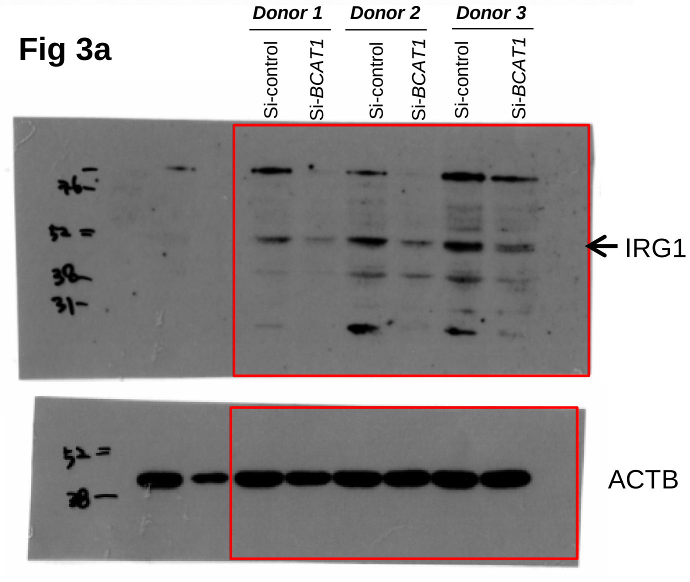

Fig 3b

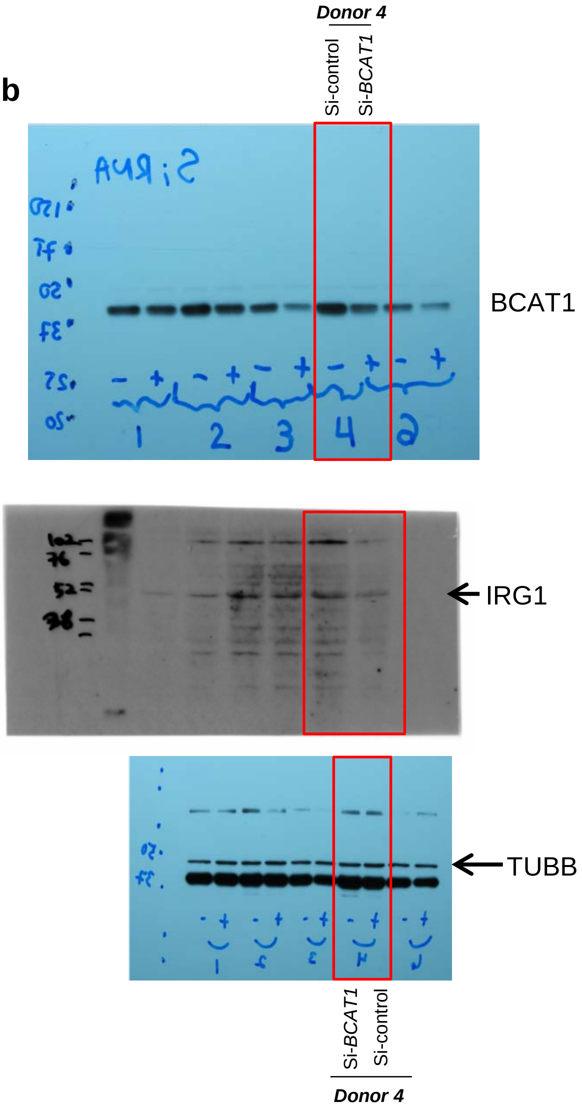

Fig 4a

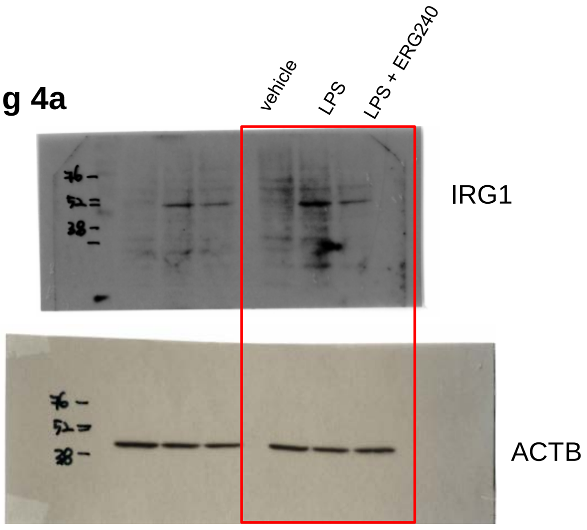

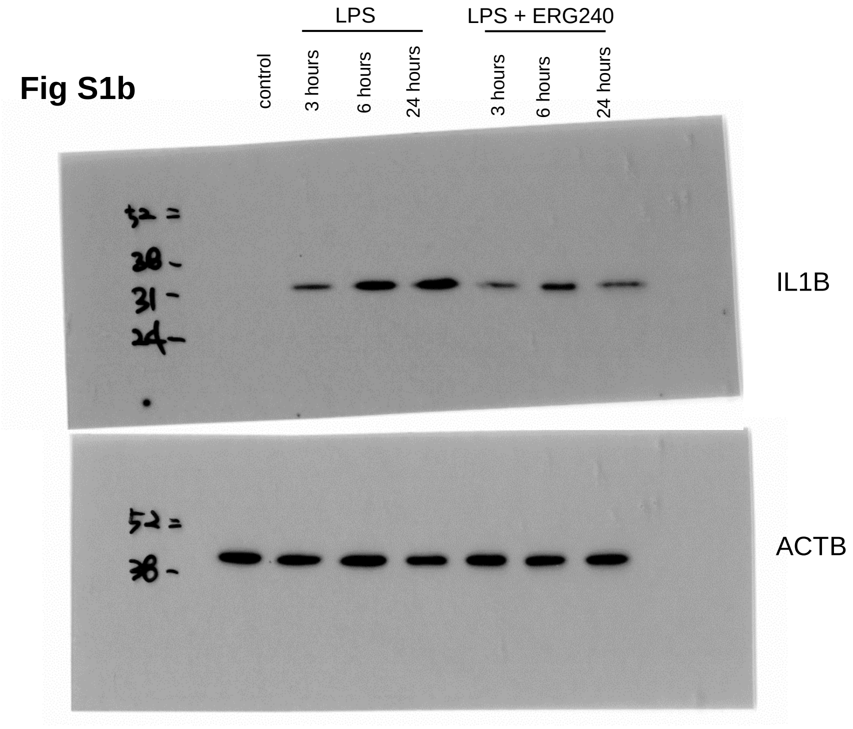

Supplementary Figure 8. Uncropped scans of the Western blots shown in the indicated figures.

Supplementary Methods

BCAT1 Enzymatic Assay

Reagents used in the BCAT1 enzymatic assays (L-leucine, pyridoxal 5’-phosphate (PLP), ammonium sulfate, dithiothreitol, NADH, a ketoglutarate, and potassium sulfate) were purchased from Sigma Aldrich. Leucine hydrogenase was obtained from EMD Millipore. Recombinant human BCAT1 and BCAT2 were from Abcam. The assay was performed as described by Copper et al. [1] with few modifications using 100 ng of recombinant protein.

RNA-sequencing library preparation

RNA was purified and fragmented using poly-T oligo-attached magnetic beads using two rounds of purification followed by the first and second cDNA strand synthesis. Next, cDNA 3' ends were adenylated and adapters ligated followed by 15 cycles of library amplification. Finally, the libraries were size selected using AMPure XP Beads (Beckman Coulter) purified and their quality was checked using Agilent 2100 Bioanalyzer. Samples were randomized to avoid batch effects and multiplexed libraries were run on a single lane (6 samples/lane) of the HiSeq 2500 platform (Illumina) to generate 100bp paired-end reads. An average of 64M and 79M reads coverage per samples was achieved for human and mouse macrophages respectively. RNA-seq reads were aligned to human (hg19) or mouse (mm10) reference genomes using tophat2. The average mapping percentage was > 90% for all samples. Sequencing and mapping were quality controlled using standard tools provided in the fastQC software. Gene level read counts were computed using HT-Seq-count with ‘union’ mode. Expression levels (in FPKM) were measured with Cufflinks 2.1.1. Protein-protein interactions were visualised using the he STRING database (http://string-db.org) as described in [2].

Migration of BMDMs and *viability of hMDMs*

The migration of BMDMs was assessed using a modified Boyden chamber methodology. Specifically, mouse BMDMs were trypsinized and suspended in serum free incomplete media containing various concentrations of the drug at a cell density of 2.5×10^4 cells/mL. Volumes of 400 μL of cell suspensions were added onto the upper chamber of 6.5 mm transwell inserts (Costar, Cat. No. 3422), placed in a 24-well plate (Costar). A volume of 1.5 mL of complete media containing 10% FBS as chemo-attractant were added into the lower chambers of the plate. The assay also included wells with lower chambers containing serum free incomplete media as negative control. Cells were allowed to migrate through the 8 μm porous polycarbonate membrane of the transwell inserts for 10 hours at 37ºC, 5% CO_2. At the end of the incubation period, migrated cells were fixed with 3.7% paraformaldehyde for 10 min, washed 3x with PBS, and stained with 0.05% crystal violet for 30 min. After washing the stained membranes 3x with PBS, migrated cells were counted using light microscopy.

To determine the effect of ERG240 on the total number of BMDMs, cells were plated onto a 96-well plate at a density of 5,000 cells per well in complete media and allowed to adhere overnight. The cells were then exposed to various drug concentrations overnight and the cell population was quantified with CyQuant Cell Proliferation kit (Life Technologies) using triplicates, in two independent experiments. For hMDMs, cell viability was determined using the alamarBlue® Assay (Invitrogen). 2×10^5 cells were stimulated with LPS, ERG240 or both for different periods (3h, 8h, 24h) and the viability was assessed at the end of each stimulation by further incubating the cells with alamarBlue solution (1/10) for 1 hour at 37˚C and monitoring absorbance at 570nm.

Clinical and histological evaluation methods in CIA

Development of arthritis was assessed every other day and scored on a scale of 0-4 as follows: 0=no oedema or swelling, 1=slight oedema and erythema restricted to the foot or ankle, 2=moderate to severe oedema and erythema restricted to the foot or ankle, 3=oedema and erythema of the entire paw, and 4=maximum inflamed limb involving multiple joints. The maximal arthritic score or arthritic index (AI) per animal was 16 (cumulative for 4 limbs). Paw thickness was measured before termination of the experiment.

In vivo pharmacokinetics

ICR mice that have been fasted overnight were administered 125 mg/kg ERG240 i.v. or 500 mg/kg ERG240 p.o. At predetermined time points, mice were sedated under general inhalant anaesthesia (3 % isoflurane) and 300-400 μL of blood were collected in tubes coated with lithium heparin by cardiac puncture. The plasma was harvested after centrifugation and kept frozen at -20 °C until further processing. Proteins present in the plasma were precipitated by acetonitrile. Specifically, 50 μL of plasma samples were transferred to a 96-well plate and 10 μL of a acetonitrile/water 50/50 (v/v) solution was added to each well. At this point, 150 μL of 1 ng/μL of aspirin in acetonitrile were also added as internal standard. After sample mixing and centrifugation, 100 μL supernatant were withdrawn from each well and mixed with 500 mL water for HPLC-MS/MS analysis. Sample analysis by HPLC-MS/MS employed a Agilent Poroshell 120 EC-C18 column 2.7μm (3.0 x 50mm) and a gradient elution with two mobile phases (A: acetonitrile/water/formic acid 99/1/0.02 (v/v/v); B:acetonitrile/water/formic acid 1/99/0.02 (v/v/v)). A volume of 10 mL of sample were injected into the column and the sample concentration was determined by employing calibration curves that were generated prior to sample analysis by spiking drug-free plasma with specified concentrations of ERG240. The quantitation range was 5-10000 ng/mL.

Following sample analysis, plots of plasma concentration of ERG240 vs. time were constructed. Fundamental pharmacokinetic parameters were calculated from the non-compartmental analysis of the plasma data using WinNonlin.

Histological evaluation of CIA tissues.

Dosing of the animals in the prophylactic groups started on the day of the booster injection (day 21), while dosing of the animals in the therapeutic groups started after arthritis was established (day 26). Animals were scored for the presence of arthritis every other day and weighed once a week. At the end of the study, sera were obtained by cardiac puncture and paws were excised for histological examination (HistoTox Labs Inc). From each therapeutic group, three front and four hind paws were selected for histological analysis. Paws were fixed in 4% formalin and decalcified in formic acid. Parafin sections (8 μm) were stained with toluidine blue (T-blue). The sections were evaluated blindly and scored for inflammation, pannus infiltration, cartilage damage, and bone erosion according to scale shown here. *Inflammation*: 0=Normal; 1=Minimal infiltration of inflammatory cells in synovium and periarticular tissue of affected joints; 2=Mild infiltration of inflammatory cells, restricted to affected joints; 3=Moderate infiltration of inflammatory cells with moderate edema, restricted to affected joints; 4=Marked infiltration of inflammatory cells affecting most areas with marked edema; 5=Severe diffuse infiltration of inflammatory cells with severe edema. *Pannus*: 0=Normal; 1=Minimal infiltration of pannus in cartilage and subchondral bone; 2=Mild infiltration of pannus in cartilage with marginal zone destruction of hard tissue in affected joints; 3=Moderate infiltration of pannus in cartilage with moderate hard tissue destruction in affected joints; 4=Marked infiltration of pannus in cartilage with marked destruction of joint architecture, most joints; 5=Severe infiltration associated with total or near total destruction of joint architecture, all joints. *Cartilage Damage*: 0=Normal;

1=Minimal to mild loss of toluidine blue staining with no obvious chondrocyte loss or collagen disruption in affected joints; 2=Mild loss of toluidine blue staining with focal mild (superficial) chondrocyte loss and/or collagen disruption in affected joints; 3=Moderate loss of toluidine blue staining with multifocal moderate (depth to middle zone) chondrocyte loss and/or collagen disruption in affected joints; 4=Marked loss of toluidine blue staining with multifocal marked (depth to deep zone) chondrocyte loss and/or collagen disruption in most joints; 5=Severe diffuse loss of toluidine blue staining with multifocal severe (depth to tide mark) chondrocyte loss and/or collagen disruption in all joints. *Bone Resorption*: 0=Normal; 1=Minimal areas of resorption not apparent on low magnification and rare presence of osteoclasts in affected joints, 2=More areas of resorption not apparent on low magnification and presence of osteoclasts in affected joints; 3=Obvious resorption of medullary trabecular and cortical bone without full thickness defects in cortex, loss of some medullary trabeculae, lesions apparent on low magnification, and presence of more osteoclasts in affected joints; 4= Full thickness defects in cortical bone, often with distortion of profile of remaining cortical surface, marked loss of medullary bone, and numerous osteoclasts in most joints; 5= Full thickness defects in cortical bone and destruction of joint architecture of all joints.

Determination of anti-CII antibody levels

Microtiter plates were coated with 100 μL of a 5 μg/mL bovine type II collagen solution from Chondrex according to the manufacturer’s instructions. Coated wells were blocked with ELISA SUNBLOCK from AbD Serotec for 1 hr and incubated with 100 μL of 1:5 diluted CIA sera. Bound IgG was detected by incubation with alkaline phosphate-conjugated rat anti-mouse IgG from KPL, followed by addition of substrate dinitrophenyl phosphate or pNPP from Southern Biotech. Optical densities were measured at 405 nm.

Supplementary References

[1] Cooper AJ, Conway M, Hutson SM. A continuous 96-well plate spectrophotometric assay for branched-chain amino acid aminotransferases. Anal Biochem, 2002;308:100-5.

[2] Szklarczyk D, Franceschini A, Wyder S, Forslund K, Heller D, Huerta-Cepas J *et al.* STRING v10: protein-protein interaction networks, integrated over the tree of life. Nucleic acids research, 2015;43:D447-52.
